# Comparative performance of PCR using DNA extracted from dried blood spots and whole blood samples for malaria diagnosis: a meta-analysis

**DOI:** 10.1038/s41598-021-83977-5

**Published:** 2021-03-01

**Authors:** Aongart Mahittikorn, Frederick Ramirez Masangkay, Kwuntida Uthaisar Kotepui, Giovanni De Jesus Milanez, Manas Kotepui

**Affiliations:** 1 Department of Protozoology, Faculty of Tropical Medicine, Mahidol University, Bangkok, Thailand; 2 Department of Medical Technology, Institute of Arts and Sciences, Far Eastern University-Manila, Manila, Philippines; 3 Medical Technology, School of Allied Health Sciences, Walailak University, Tha Sala, Nakhon Si Thammarat, Thailand

**Keywords:** Malaria, Diagnostic markers

## Abstract

Polymerase chain reaction (PCR) using deoxyribonucleic acid (DNA) extracted from dried blood spots (DBS) provides a fast, inexpensive, and convenient method for large-scale epidemiological studies. This study compared the performance of PCR between DNA extracted from DBS and DNA obtained from whole blood for detecting malarial parasites. Primary studies assessing the diagnostic performance of PCR using DNA extracted from DBS and whole blood for detecting malarial parasites were obtained from the ISI Web of Science, Scopus, and PubMed databases. Odds ratios (ORs) and 95% confidence intervals (CIs) were plotted in forest plots using Review Manager version 5.3. Statistical analysis was performed via random-effects meta-analysis. Data heterogeneity was assessed using the *I*^2^ statistic. Of the 904 studies retrieved from the databases, seven were included in this study. The pooled meta-analysis demonstrated no significant difference in the comparative performance of PCR for detecting malaria parasites between DNA extracted from DBS and that extracted from whole blood (OR 0.85; 95% CI 0.62–1.16; *I*^2^ = 78%). However, subgroup analysis demonstrated that PCR using DNA extracted from DBS was less accurate in detecting *Plasmodium vivax* than that using DNA extracted from whole blood (OR = 0.85; 95% CI 0.77–0.94). In conclusion, a significant difference in detecting *P. vivax* was observed between PCR using DNA extracted from DBS and that using DNA extracted from whole blood. Therefore, *P. vivax* in endemic areas should be identified and detected with care with PCR using DNA obtained from DBS which potentially leads to a negative result. Further studies are required to investigate the performance of PCR using DBS for detecting *P. vivax* and other malarial parasites to provide data in research and routine surveillance of malaria, especially with renewed efforts towards the eradication of the disease.

## Introduction

The gold standard method for detecting malarial parasites in routine laboratories is based on the examination of thick and thin blood films under light microscopy^1^. The microscopic method provides several advantages for the diagnosis of malarial parasites including a relatively simple technique, low cost, and the ability to identify *Plasmodium* species and its parasite density; however, the microscopic method provides a low limit of detection (LOD) of malarial parasites with a sensitivity of 50–500 parasites/μl^2^. Another malarial detection method is the use of rapid diagnostic tests (RDTs) that detect malarial antigens in the blood of patients with a sensitivity of ~ 100 parasites/μl^2^. The commonly targeted RDT antigens are histidine-rich protein 2 (HRP2), lactate dehydrogenase (pLDH), and aldolase, among which HRP2 is the most commonly used target in commercial kits due to its specificity to *P. falciparum*^3^. Although RDTs have been used in remote parts of malaria-endemic areas, they present several disadvantages like false positives in the blood of patients who were cured of malaria infections^4^. Also, RDTs can provide false-positive results for non-*P. falciparum* malaria if patients have a high-parasite density of *P. falciparum*^5^. RDTs can provide false-negative results due to gene deletions^6^ or a low parasite density of malarial parasites in the blood samples^3^. The introduction of molecular assays such as polymerase chain reaction (PCR) has improved the sensitivity and specificity of malarial diagnosis^7–9^. Molecular assays have allowed the detection of submicroscopic *Plasmodium* infection in parasite carriers with high sensitivity as well as the identification of all five malarial species^10,11^. Therefore, PCR is particularly valuable when subjective microscopy does not permit the identification of certain malarial species. Moreover, it can indicate monoinfections and mixed infections and serves as a useful tool for the epidemiological understanding of malarial infections. The principle of PCR is to amplify a targeted malarial DNA in whole blood samples via venipuncture or finger-prick. Although blood collection using a standard venipuncture technique provides a large amount of blood, it has several limitations including patient reluctance, transportation of blood samples, and prolonged freezing of blood, making it difficult to conduct malarial epidemiological surveys among patients or residents in resource-limited settings. To overcome the limitations of venous blood collection, dried blood spots (DBSs) were introduced as a blood collection technique for a minute amount of blood. DBSs require a small amount of blood to be dropped onto a filter paper which is collected using the finger-prick method that is less invasive than venous blood collection^12^. Therefore, it is a blood collection technique that is conveniently performed in large-scale epidemiological studies^13,14^. Although DBS has advantages over venous blood collection in large-scale studies and surveys undertaken in remote areas, several studies have demonstrated that the PCR method using DNA extracted from DBS had a lower sensitivity than PCR using DNA extracted from venous blood collection^14–17^. Because the collection and transport of blood samples are critical for studies targeting malarial parasites, assessing the performance of PCR using extracted DNA from DBS and whole blood is critical for providing confidence in using DBS, which is currently used in different studies. Thus, this study assessed the performance of PCR using DNA from DBS and whole blood for detecting malarial parasites in patients. The results of this study will determine the advantages and disadvantages of using DBS, which are essential considerations in research and routine surveillance of malaria, especially with renewed efforts toward the eradication of the disease.

## Methods

This systematic review and meta-analysis followed the Preferred Reporting Items for Systematic Reviews and Meta-Analyses (PRISMA) guidelines^18^.

### Eligible studies

Primary studies that assessed the diagnostic accuracy of PCR using DNA obtained from both DBS and whole blood for the detection of malaria parasites were eligible. The inclusion criteria were as follows: studies including febrile patients seeking care at health facilities or residents in communities in malaria-endemic regions and studies comparing the performance of PCR using DNA from DBS and whole blood to confirm cases of malaria. Animal studies, case reports, case series, clinical drug trials, experimental studies, vector studies, polymorphism studies, short reports, and reviews were excluded, as well as studies not written in the English language, those without full text, and those with incomplete data for extraction.

### Search strategy

The search strategy began with electronic databases including ISI Web of Science, Scopus, and PubMed with the provided search terms (Table S1). Literature searches were started and ended at 4 March 2020. To avoid missing studies, the search terms were kept broad and provided as ‘dried blood spot’ AND (‘polymerase chain reaction’ OR PCR) AND (malaria OR *Plasmodium*). In addition, the reference lists of the selected studies, reviews, and systematic reviews were manually checked for other possible related studies.

### Selection criteria

The selection criteria were based on title and abstract selections by two independent authors (AM and MK). Any duplicate studies were removed by Endnote software (Clarivate Analytics). Studies that were not related to *Plasmodium* species or malaria and those in which PCR was not used for detection were removed following title and abstract screening. All studies considered relevant to the eligibility criteria were selected, and the full text was evaluated. Disagreements between the two reviewers were resolved by consulting a third author (GM or FM). Study information and fulfilment of the inclusion criteria were recorded in Microsoft Excel spreadsheets (Microsoft Corporation, CA, USA) for further analysis.

### Data extraction

Two authors (AM and MK) selected and extracted data from the included studies independently into Microsoft Excel spreadsheets. The extracted information was as follows: authors, year of publication, participants, age range, gender, blood storage for PCR, DNA extraction methods, investigated gene, and the results of PCR using both filter paper and whole blood.

### Statistical analysis and data synthesis

A meta-analysis was performed to assess the comparative performance of PCR using DNA extracted from DBS and DNA extracted from whole blood for malarial detection. Odds ratios (ORs) and 95% confidence intervals (CIs) were plotted in forest plots using Review Manager (RevMan) Version 5.3 (The Cochrane Collaboration). Statistical analysis was performed using random-effects meta-analysis if unexplained heterogeneity was frequently present. Subgroup analysis of *Plasmodium* species was performed. Data heterogeneity was assessed using the *I*^2^ statistic, and values exceeding 25% indicated moderate or high heterogeneity among the included studies.

### Quality of the included studies

The quality of included studies was assessed using the Newcastle–Ottawa scale (NOS) for assessing the quality of nonrandomized studies in meta-analyses^19^, and the highest score was eight stars.

### Publication bias

The publication bias of the included studies was assessed by funnel plot symmetry.

## Results

### Characteristics of the included studies

**Figure 1:**
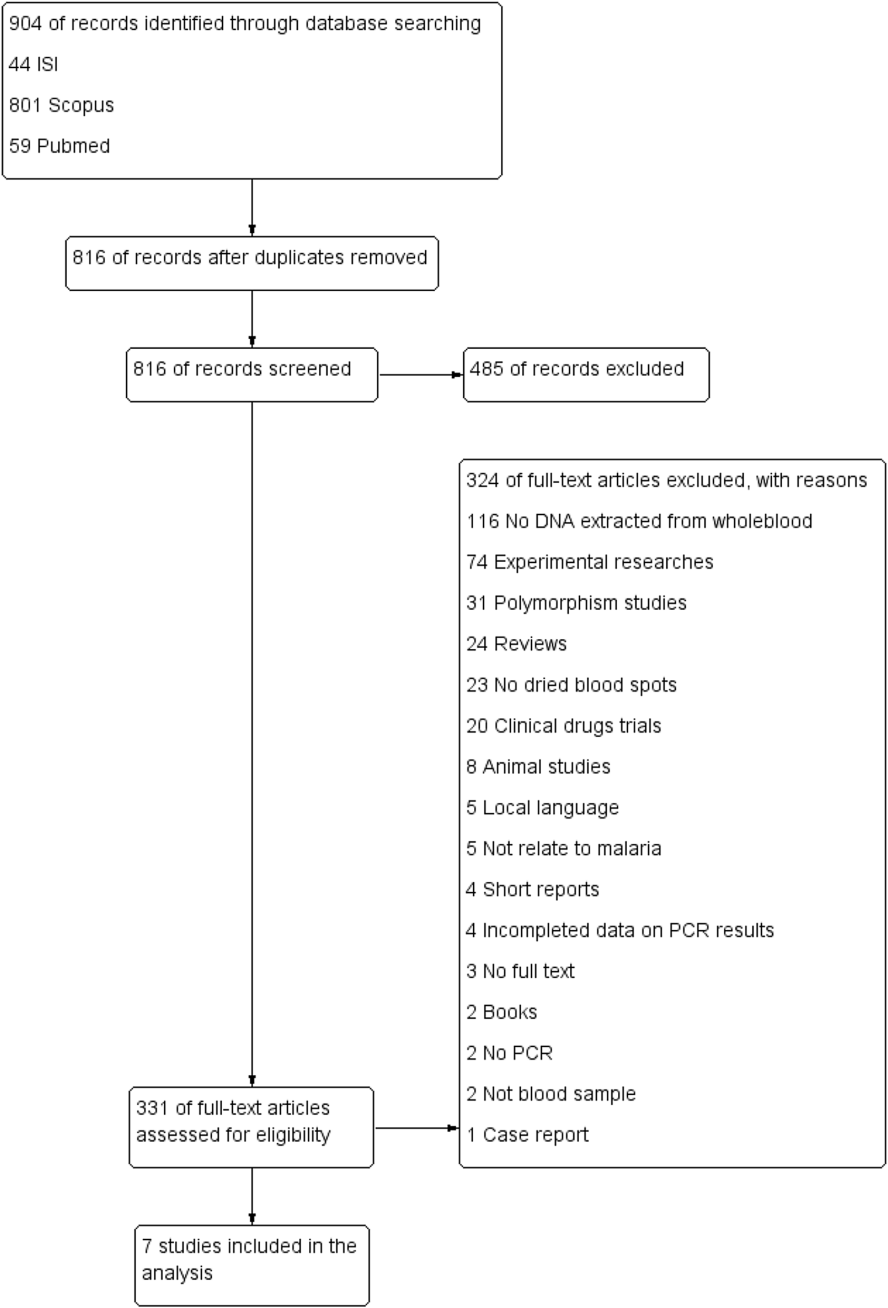
Flow chart for study selection.

The searches of PubMed, ISI Web of Science, and Scopus databases retrieved 59, 44, and 801 studies, respectively. After removing duplicates, 816 studies were screened using the title and abstract. Then, the full text of 331 studies was screened. Of these studies, 324 were excluded for several reasons, as detailed in Fig. 1. Finally, seven studies^9,12,14–16,20,21^ were included in the present study (Table 1). All included studies were published between 2008 and 2017, and 4001 malaria positive-patients as identified using DNA extracted from DBS and 3878 malaria positive-patients as identified using DNA extracted directly from whole blood were included. Most of the included studies (6/7, 85.7%) were conducted in Asian countries including Saudi Arabia^15^, Iran^14^, Cambodia^9^, Thailand^16^, Canada^20^, and Myanmar^21^, whereas one study was conducted in Tanzania^12^. Among 7 studies, 3 studies^12,15,16^ (42.9%) recruited only febrile participants for the experiments. Five studies^12,14,16,20,21^ did not specify the age range and four studies^12,16,20,21^ did not specify the gender of patients in their studies. The Chelex extraction method was used to extract DNA from DBS in 3 studies (42.9%)^12,16,20^, whereas the 4 studies^9,14,15,21^ used a QIAamp DNA blood mini kit to extract DNA from whole blood (5/7, 71.4%). Six studies^12,14–16,20,21^ used nested PCR to amplify the malarial *18S rRNA* gene in their studies, whereas only one study^9^ used nested PCR to amplify the malarial *cytochrome b* gene. In the present study, we attempted to perform subgroup analysis even though only a few included studies were selected to explore the association between the study characteristics and the primary outcome of this study.

**Table 1:** Characteristics of the included studies.

No	Author, year	Study area (years of the survey)	Participants	Age range	Gender	Blood storage for PCR	Blood spot on filter paper	DNA extraction, PCR from filter paper	Whole blood	DNA extraction, PCR from whole blood	Investigated gene	PCR method	PCR method
1	Al-Harthi and Jamjoom, 2008	Saudi Arabia (2005–2006)	Febrile (118)	2 months to more than 50 years	Males = 83 Females = 35	4 °C and cool container	Whatman filter papers, four drops	QIAamp mini kit (68, 57.6%) Methanol fixation heat-Extraction method (49, 41.5%)	NA	QIAamp DNA blood mini kit (79, 67%)	*18 S rRNA*	Nested PCR	Ciceron et al., 1999
2	Ataei et al., 2011	Iran	Febrile (45) Positive (15) Negative control (15)	NA	Males = 59 Females = 16	Frozen liquid blood specimens	DBC filter paper, three drops of finger-prick blood	Kawsar Genomics & Biotech Center kit (Pv = 26, Pf = 6)	200 µL	Bioneer Accupreps Genomic DNA extraction kit (Pv = 29, Pf = 6)	*18 S rRNA*	Semi-nested multiplex PCR	Rubio et al., 2002
3	Canier et al., 2015	Cambodia (2013)	Residents (521)	< 5 years (44) > 5 years (477)	Males = 222 Females = 299	4 °C and cool container	3-mm Whatman filter, 5-µL aliquot	Bio-Rad Instagene matrix (Pv = 41, Pf = 8)	200 µL	QIAamp DNA blood mini kit (Pv = 54, Pf = 11)	*Cytochrome b*	Real-time PCR assays	NA
4	Proux et al., 2011	Thailand	Febrile (413)	NA	NA	Room temperature (12 h) in the laboratory in Mae-Sot, frozen to Paris	Whatman 3-mm filter paper, 30-µL aliquot	Chelex extraction (Pv = 175, Pf = 170, mixed = 43)	5 µL	QIAamp DNA blood mini kit (Pv = 174, Pf = 163, mixed = 29)	*18 S rRNA*	Nested PCR	Snounou et al., 2002
5	Strøm et al., 2014	Tanzania (2009)	Febrile (469)	Children	NA	25 °C for 3–9 months, storage for approximately 3.5 years at − 20 °C until DNA extraction	Whatman Schleicher & Schuell filter paper, two drops	Chelex-100 Molecular Biology Grade Resin (Pf = 52/442, 11.8%)	200 µL	QIAamp DNA blood mini kit (Pf = 78/319, 24.5%)	*18 S rRNA*	Nested PCR	Haanshuus et al., 2013
6	Taylor et al., 2011	Canada (2008–2011)	Febrile (67) Asymptomatic patients (25) Negative (7) *Plasmodium knowlesi* (1)	NA	NA	− 20 °C and thawed at 4 °C prior to testing	Whatman 3-mm filter paper, 1.5 mm diameter blood spots	Chelex 100 (Sigma) (74/100, 74%)	NA	Chelex 100 (69/100, 69%)	*18 S rRNA*	Real-time PCR	Kamau et al., 2011
7	Zainabadi et al., 2017	Myanmar (2015)	Residents (2332)	NA	NA	Room temperature (7–10 months)	Whatman 3-mm filter paper, 50-µL aliquot	Ultrasensitive method (Pv = 950, Pf = 249, mixed = 9)	300 µL	QIAamp DNA extraction method (Pv = 1055, Pf = 229, mixed = 12)	*18 S rRNA*	RT-PCR	Adams et al., 2015

*Pv* *Plasmodium vivax*, *Pf* *Plasmodium falciparum*, *mixed* mixed infection, *NA* Not Assessed.

### Quality of included studies

All seven included studies were given scores of eight stars on the NOS based on their fit to the inclusion and exclusion criteria (Table 2).

**Table 2:** Quality of the included studies.

No	Reference	Selection				Compatibility	Exposure			Total score (8)
		Is the case definition adequate?	Representativeness of the cases	Selection of controls	Definition of controls		Ascertainment of exposure	Same method of ascertainment for cases and controls	Non-response rate	
1	Al-Harthi and Jamjoom, 2008	*	*	*	*	*	*	*	*	8
2	Ataei et al., 2011	*	*	*	*	*	*	*	*	8
3	Canier et al., 2015	*	*	*	*	*	*	*	*	8
4	Proux et al., 2011	*	*	*	*	*	*	*	*	8
5	Strøm et al., 2014	*	*	*	*	*	*	*	*	8
6	Taylor et al., 2011	*	*	*	*	*	*	*	*	8
7	Zainabadi et al., 2017	*	*	*	*	*	*	*	*	8

* A star rating

### Comparative performance of PCR using DNA extracted from DBSs and whole blood samples

**Figure 2:**
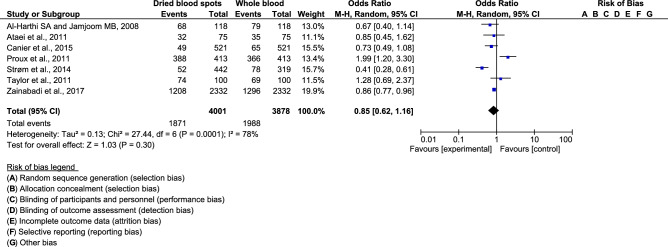
Forrest plot demonstrated the performance of polymerase chain reaction using deoxyribonucleic acid from dried blood spots and whole blood.

Regarding the results of individual studies, two studies illustrated that DNA extracted from DBS had lower performance for detecting malaria parasites (OR 0.41, 95% CI 0.28–0.61; OR 0.86, 95% CI 0.77–0.96)^12,21^, whereas one study demonstrated that DNA from DBS had higher performance (OR 1.99, 95% CI 1.20–3.30)^16^. The pooled meta-analysis revealed no significant difference in the comparative performance of PCR for detecting malaria parasites between using DNA from DBS and whole blood (OR 0.85, 95% CI 0.62–1.16, P = 0.3). There was high heterogeneity among the included studies (*I*^2^ = 78%, P = 0.0001; Fig. 2).

### Subgroup analysis

**Figure 3:**
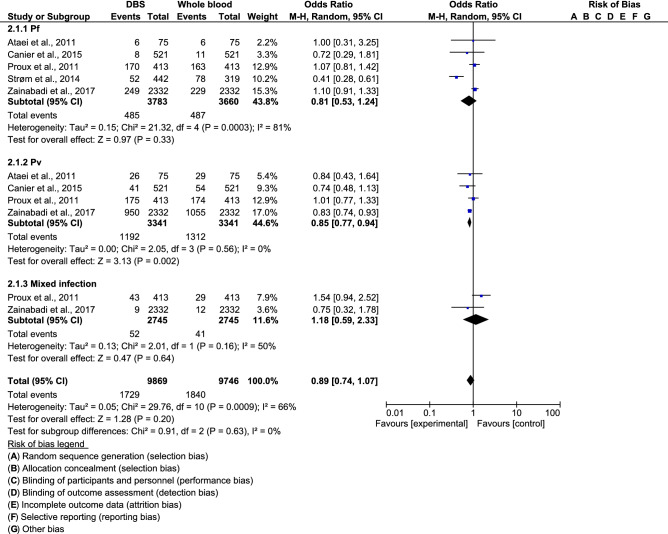
Subgroup analysis of *Plasmodium* species and the performance of polymerase chain reaction using deoxyribonucleic acid from dried blood spots and whole blood.

Subgroup analysis demonstrated that PCR using DNA extracted from DBS was lower in performance for detecting *Plasmodium vivax* than PCR using DNA extracted from whole blood (OR 0.85, 95% CI 0.77–0.94, P = 0.002). No difference was noted in the detection of *P. falciparum* or mixed infection between DBS and whole blood (Fig. 3).

### Publication bias

**Figure 4:**
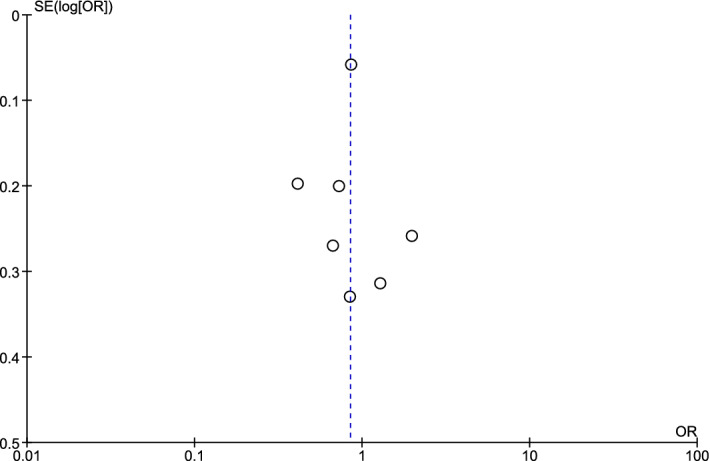
Funnel plot demonstrating publication bias among the included studies.

Publication bias was assessed in the present study using a funnel plot. The symmetry of the plot indicated that no publication bias was present (Fig. 4).

## Discussion

In the present study, the performance of PCR using DNA from DBS and whole blood was compared. The results of the meta-analysis of 7 studies showed no difference in the detection of malaria parasites between the two blood samples for PCR analysis. As the results of the meta-analysis were heterogeneous, one-by-one or subgroup analysis was required to determine the sources of heterogeneity among the included studies. One by one interpretation of the included studies was performed. The study by Proux et al. showed that PCR using DNA extracted from DBS had a greater two-fold performance for detecting malarial parasites than PCR using DNA extracted from whole blood^16^. Although the study of Proux et al. indicated that DBS had greater sensitivity for detecting malaria parasites, the experiments were performed in different laboratories with different reagents, thermocyclers, and technicians^16^; therefore, the higher performance of PCR using DNA extracted from DBS than PCR using DNA extracted from whole blood requires further investigation. A study by Strom et al. has demonstrated the lower performance of PCR using DNA extracted from DBS than PCR using DNA extracted from whole blood^12^.

The subgroup analysis of this study demonstrated that PCR using DNA extracted from DBS provided a lower performance in detecting *P. vivax* than PCR using DNA extracted from whole blood, and no difference in performance for detecting *P. falciparum* or mixed infections was observed. Although no difference was demonstrated between the comparative performance of PCR using DNA extracted from DBS and PCR using DNA extracted from whole blood for detecting *P. falciparum* when using the results of the five included studies, the study by Strom et al. has demonstrated a lower performance of PCR using DNA extracted from DBS and suggested that the concentration of DNA in DBS was lower than that in whole blood, and PCR using DNA extracted from DBS missed 46.1% of *P. falciparum* infections^12^. For the performance of PCR in detecting *P. vivax* malaria, the result of the meta-analysis of four studies demonstrated that PCR had a lower efficiency using DNA extracted from DBS than PCR using DNA extracted from whole blood. In addition, no heterogeneity was observed among the four included studies, and therefore, the results of this analysis can be interpreted reliably. Among the four included studies, the study by Canier et al. has demonstrated that PCR using DBS missed 27% of malarial parasites, mostly *P. vivax* (84%)^9^. In addition, a study by Ataei et al. demonstrated that PCR using DBS missed only 10.3% of *P. vivax* infections^14^. Furthermore, a study by Zainabadi et al. indicated that PCR using DBS missed 10% of *P. vivax* infections and 25% of mixed infections^21^.

A previous study indicated that the lower performance of PCR using DBS compared to using whole blood might be attributable to the DNA extraction methods, the type of filter paper, amplification factors, and sample storage^15,17,22^. Only two included studies, namely those by Al-Harthi et al. and Taylor et al., used the same DNA extraction method for both DBS and whole blood^15,20^. The study by Al-Harthi et al. demonstrated that the DNA extracted using a commercial kit provided a higher quality of DNA than the conventional methanol fixation method^15^. Despite the use of the same DNA extraction method (QIAamp DNA blood mini kit), the sensitivity of PCR was lower for DBS (57.6%) than for whole blood (67%)^15^. The study by Taylor et al. used Chelex 100 (Sigma) for DNA extraction for both DBS and whole blood. However, they performed real-time PCR, which displayed higher sensitivity for malaria detection from DBS (74%) than from whole blood (69%)^20^. This study used different PCR conditions for the two sample types, including differences in the number of PCR cycles and cycle threshold to overcome the high background fluorescence of the filter paper^20^.

A study has demonstrated that DBS contained lower quantities of malarial DNA for amplification resulting in lower detection even after PCR, whereas whole blood samples contain robust quantities of malarial DNA for more successful DNA amplification regardless of the PCR method^12,16^. Therefore, the lower efficiency of PCR using DNA extracted from DBS for detecting *P. vivax* might be due to the low quantity and quality of DNA. Other factors that might contribute to the low performance of PCR using DNA extracted from DBS include, but not limited to, an extremely low level of parasitemia^16^, high temperature^12^, and humidity^17,23^, which might negatively influence the overall quality of DBS samples during storage. Relative to this, exploring and defining optimal storage protocols and conditions for DBS samples for improved recovery of malarial DNA using molecular methods seem beneficial. Despite the lower efficiency of PCR using DNA extracted from DBS, it allowed the detection of malarial parasites in several samples that were negative on microscopic examination. Moreover, DBS offers many advantages including the use of finger-prick sampling, long-term storage at room temperature, easy sample transport, and simple and rapid protocols for diagnostic purposes.

This study had some limitations. First, a small number of studies comparing PCR using whole blood with that using DBS were included in the analysis. Second, the included studies vary in the protocol of DNA extraction methods and PCR protocols and, therefore, might be the source of heterogeneity across the included studies. Third, as the results of the meta-analysis were heterogeneous, the comparative performance of PCR using DNA extracted from DBS compared with PCR using DNA extracted from whole blood must be interpreted carefully. In conclusion, the comparative performance of PCR using DNA extracted from DBS and PCR using DNA extracted from whole blood was significantly different in detecting *P. vivax*. Therefore, detecting *P. vivax* in endemic areas should be interpreted with care with PCR in cases where PCR using DNA extracted from DBS potentially gives a negative result. Further studies are required to improve the use of DBS for molecular methods possibly by defining the optimal storage protocols and conditions to protect the integrity of the low quantities of DNA. This will enhance the detection of *P. vivax* and other malarial parasites, which will be helpful in conducting studies and performing routine surveillance of malaria, especially with renewed efforts toward the eradication of the disease.

## Supplementary Information

Supplementary Information
