# Supplementary material for: Comparative performance of PCR using DNA extracted from dried blood spots and whole blood samples for malaria diagnosis: a meta-analysis
Source: Sci Rep. 2021 Mar 1;11:4845. doi: 10.1038/s41598-021-83977-5 (PMC7921648; doi:10.1038/s41598-021-83977-5)
Supplement: Supplementary file 1 — Supplementary Information [file 41598_2021_83977_MOESM1_ESM.docx]

**Comparative performance of PCR using DNA extracted from dried blood spots and whole blood samples for malaria diagnosis: A meta-analysis**

Aongart Mahittikorn ^1^, Frederick Ramirez Masangkay^2^, Kwuntida Uthaisar Kotepui^3^, Giovanni De Jesus Milanez^2^, Manas Kotepui ^3*^

^1^ Department of Protozoology, Faculty of Tropical Medicine, Mahidol University, Bangkok, Thailand

^2^ Department of Medical Technology, Institute of Arts and Sciences, Far Eastern University-Manila, Manila, Philippines

^3^ Medical Technology, School of Allied Health Sciences, Walailak University, Tha Sala, Nakhon Si Thammarat, Thailand

Authors’ Email Addresses:

**^*^Corresponding Author**: Manas Kotepui; manaskote@gmail.com

Aongart Mahittikorn; aongart.mah@mahidol.ac.th

Frederick Ramirez Masangkay; frederick_masangkay2002@yahoo.com

Kwuntida Uthaisar Kotepui; kwuntida.ut@wu.ac.th

Giovanni De Jesus Milanez; gmilanez@feu.edu.ph

**S1 Table. Search term**

| **Databases** | **Search terms** | **Search date** |
| --- | --- | --- |
| MEDLINE | "Dried blood spot"[All Fields] AND ("Polymerase chain reaction"[All Fields] OR PCR[All Fields]) AND (("malaria"[MeSH Terms] OR "malaria"[All Fields]) OR ("plasmodium"[MeSH Terms] OR "plasmodium"[All Fields])) | 4 March 2020 |
| Scopus | "Dried blood spot"[All Fields] AND ("Polymerase chain reaction"[All Fields] OR PCR[All Fields]) AND (("malaria"[MeSH Terms] OR "malaria"[All Fields]) OR ("plasmodium"[MeSH Terms] OR "plasmodium"[All Fields]))  Search option: All fields | 4 March 2020 |
| ISI Web of Science | "Dried blood spot"[All Fields] AND ("Polymerase chain reaction"[All Fields] OR PCR[All Fields]) AND (("malaria"[MeSH Terms] OR "malaria"[All Fields]) OR ("plasmodium"[MeSH Terms] OR "plasmodium"[All Fields]))  Search option: All fields | 4 March 2020 |
